# Challenges in Implementing Artificial Intelligence in Breast Cancer Screening Programs: Systematic Review and Framework for Safe Adoption

**DOI:** 10.2196/62941

**Published:** 2025-05-15

**Authors:** Serene Goh, Rachel Sze Jen Goh, Bryan Chong, Qin Xiang Ng, Gerald Choon Huat Koh, Kee Yuan Ngiam, Mikael Hartman

**Affiliations:** 1 Department of Surgery National University Hospital Singapore Singapore; 2 Yong Loo Lin School of Medicine National University Singapore Singapore Singapore; 3 Saw Swee Hock School of Public Health National University Heart Centre Singapore Singapore Singapore; 4 National University Hospital Singapore Singapore Singapore

**Keywords:** artificial intelligence, breast cancer, breast screening, digital health, cancer screening, screening, public health, risk, mortality, radiology, mammograms, mammogram, AI, efficiency, accuracy, imaging, applications, application

## Abstract

**Background:**

Artificial intelligence (AI) studies show promise in enhancing accuracy and efficiency in mammographic screening programs worldwide. However, its integration into clinical workflows faces several challenges, including unintended errors, the need for professional training, and ethical concerns. Notably, specific frameworks for AI imaging in breast cancer screening are still lacking.

**Objective:**

This study aims to identify the challenges associated with implementing AI in breast screening programs and to apply the Consolidated Framework for Implementation Research (CFIR) to discuss a practical governance framework for AI in this context.

**Methods:**

Three electronic databases (PubMed, Embase, and MEDLINE) were searched using combinations of the keywords “artificial intelligence,” “regulation,” “governance,” “breast cancer,” and “screening.” Original studies evaluating AI in breast cancer detection or discussing challenges related to AI implementation in this setting were eligible for review. Findings were narratively synthesized and subsequently mapped directly onto the constructs within the CFIR.

**Results:**

A total of 1240 results were retrieved, with 20 original studies ultimately included in this systematic review. The majority (n=19) focused on AI-enhanced mammography, while 1 addressed AI-enhanced ultrasound for women with dense breasts. Most studies originated from the United States (n=5) and the United Kingdom (n=4), with publication years ranging from 2019 to 2023. The quality of papers was rated as moderate to high. The key challenges identified were reproducibility, evidentiary standards, technological concerns, trust issues, as well as ethical, legal, societal concerns, and postadoption uncertainty. By aligning these findings with the CFIR constructs, action plans targeting the main challenges were incorporated into the framework, facilitating a structured approach to addressing these issues.

**Conclusions:**

This systematic review identifies key challenges in implementing AI in breast cancer screening, emphasizing the need for consistency, robust evidentiary standards, technological advancements, user trust, ethical frameworks, legal safeguards, and societal benefits. These findings can serve as a blueprint for policy makers, clinicians, and AI developers to collaboratively advance AI adoption in breast cancer screening.

**Trial Registration:**

PROSPERO CRD42024553889; https://tinyurl.com/mu4nwcxt

## Introduction

Breast cancer is the most commonly diagnosed cancer worldwide, with its global prevalence expected to rise in tandem with the aging population. By 2040, projections indicate that over 3 million new cases of breast cancer will be diagnosed annually [1]. This growing global prevalence underscores the urgency of addressing the disease as a public health challenge. Many countries worldwide have embraced mammographic screening programs as a vital tool for identifying breast cancer in its early stages, significantly reducing the risk of associated mortality [2].

Despite the perceived advantages, numerous challenges remain in the interpretation of screening mammograms. First, the high volume of screenings, combined with the requirement for independent, blinded double-reading by radiologists, places significant pressure on the existing radiology workforce [3]. Second, high false-positive recall rates on initial screening often lead to additional procedures and cause undue anxiety for the patient [4]. Third, approximately 25% of cancers—known as interval breast cancers—are diagnosed between routine screening mammograms that initially appear normal and the next scheduled screening, despite adherence to regular screening intervals [5].

Artificial intelligence (AI) presents a solution by automating and streamlining these processes, potentially augmenting both efficiency and accuracy. However, the adoption of AI in breast cancer screening is not without challenges. Although there are over 20 Food and Drug Administration (FDA)–approved AI applications for breast imaging, their adoption and utilization in clinical settings remain highly variable and generally low [6]. Significant barriers to the implementation of AI in breast screening include inconsistent performance, limited generalizability of AI algorithms across diverse scenarios, and a lack of confidence among health care providers. These challenges underscore the need for well-defined frameworks to guide the implementation of AI in breast screening.

To date, there is a paucity of regulatory frameworks specific to breast cancer screening. This regulatory gap can lead to uncertainty and hesitation in adopting AI technologies in clinical practice. A comprehensive AI governance framework is critical as the medical community considers adopting AI as a second reader in screening programs. Hence, there is an urgent need to develop a holistic AI governance framework to support this ongoing transition [7,8]. It is well known that the implementation process can be influenced by various factors, including the characteristics of the institution and its broader environment, as well as the attributes of the individuals delivering the service—who are often practitioners rather than researchers [9,10]. Implementation science serves as a conduit for translating research findings into practical applications in real-world settings. To this end, various implementation theories and frameworks have been developed, each tailored to the specific goals of the research [11]. Determinant frameworks, for instance, focus on identifying barriers and facilitators (independent variables) that influence implementation outcomes (dependent variables).

The main aim of this study is to identify the challenges associated with implementing AI in breast screening programs, as highlighted in the existing literature, and to discuss a practical framework for the safe integration of AI imaging in breast cancer screening, utilizing the updated 2022 Consolidated Framework for Implementation Research (CFIR) [12]. The CFIR is a popular determinant and theoretical framework that offers a detailed taxonomy of factors influencing implementation across various socioecological levels, including community, organizational, and individual aspects. This framework is particularly applicable to the integration of AI in breast cancer screening, as it helps identify and address the diverse barriers and facilitators inherent in this complex process.

## Methods

### Search Strategy and Selection Criteria

This systematic review was registered on the International Prospective Register of Systematic Reviews (PROSPERO), registration number CRD42024553889, and is structured in line with the PRISMA (Preferred Reporting Items for Systematic Reviews and Meta-Analyses) checklist [36]. For this systematic review, we searched literature from the inception of the databases to January 30, 2024, to identify articles with guidance for the development of a governance framework for the adoption of AI in breast cancer screening. The databases searched were PubMed, Embase, and MEDLINE. Keywords used in the search were “artificial intelligence,” “regulation,” “governance,” “breast cancer,” and “screening.” The full search terms and strategy are displayed in Multimedia Appendix 1.

Two reviewers (RSJG and BC), independent of each other, evaluated the articles at both the title/abstract and full-text stages based on a priori inclusion and exclusion criteria. A third independent reviewer (SG) was consulted when a consensus could not be reached. The inclusion criteria were studies that examined the use of AI in breast cancer imaging; provided clear methodologies for assessing AI systems, including performance metrics and best practices for clinical implementation; and were published in peer-reviewed journals. AI is defined to encompass both traditional computer-assisted detection systems and modern machine learning and deep learning approaches. Excluded studies were those unrelated to AI in breast cancer detection, those published in languages other than English, abstracts, nonoriginal research, and studies lacking sufficient methodological detail.

### Data Analysis and Synthesis

Data collection was conducted in a blinded manner by 2 independent reviewers (RSJG and BC) using a predetermined data collection form. The variables collected included title, author, year, country, objective, methods, and reporting of key challenges and solutions. The findings were synthesized using a narrative synthesis approach. Initially, a preliminary synthesis was conducted through thematic analysis, which involved searching for relevant studies, listing them, and presenting the results in tabular form. Subsequently, the results were discussed and organized into themes. Finally, the included studies were summarized in a narrative synthesis within the CFIR [12], with all authors reaching a consensus. This framework comprised the following constructs: innovation characteristics (relative advantage, adaptability, complexity, and design factors), outer setting (local attitudes, policies and laws, partnerships, and connections), inner setting (infrastructure, networks, mission alignment, and available resources), and individual characteristics (knowledge, capabilities, motivation, and opportunities).

### Quality Assessment

The relevance and quality of the selected studies were assessed independently by at least two reviewers with reference to quality domains adapted from Batini et al [13] and Bano and Zowghi [14]:

- Accuracy: The objectives of the study are clearly stated, with the data collection methodology adequately described. Important statements should be supported with references.
- Consistency: The design of the study is appropriate for the research objectives. The study’s research questions are answered, or the research objective is attained.
- Completeness: The study’s research approach is described in sufficient detail.
- Timeliness: The study was published within the past 10 years.
- Relevance: An additional domain was included to ensure that the study was relevant and provided substantial discussion evaluating the use of AI in breast cancer screening.

The studies were assessed in the respective domains on a scale of 1-5, with 1 representing minimal relevance in the domain and 5 representing high relevance in the domain. The quality of the studies was assessed by 2 independent reviewers (RSJG and BC), with a third author (SG) resolving any disagreements.

## Results

### Study Inclusion and Quality

**Figure 1:**
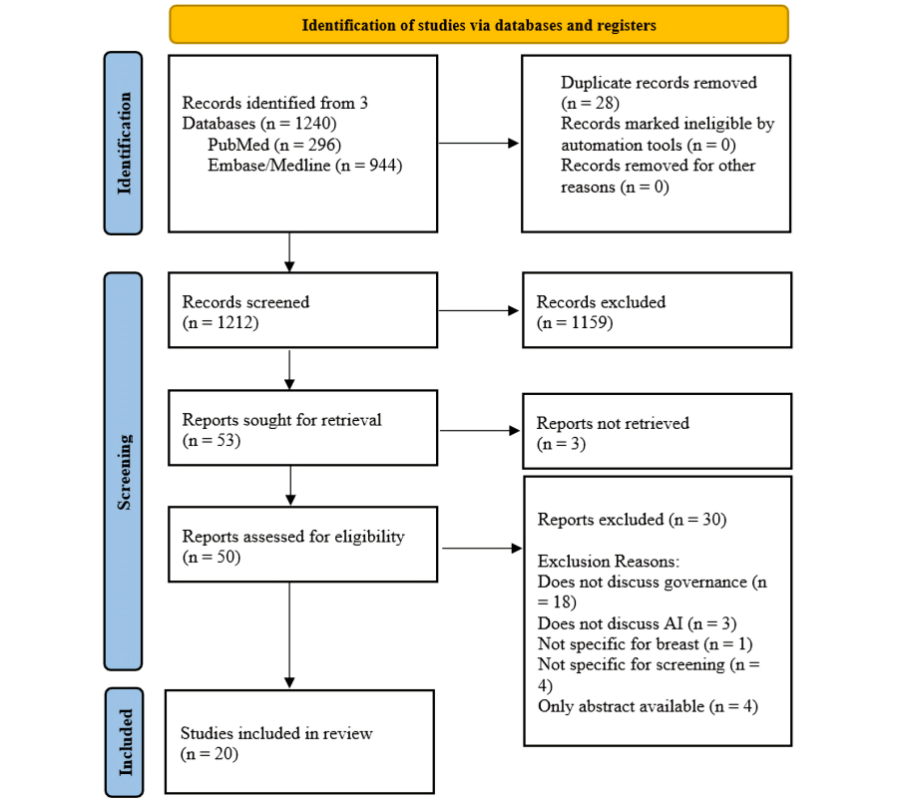
PRISMA flowchart.

A total of 1240 abstracts were retrieved from the databases for the initial sieve. After 28 duplicates were removed, a priori inclusion and exclusion criteria were applied at 2 stages: the title/abstract sieve and the full-text sieve. A total of 20 articles were included in this systematic review, with the abstraction process shown in Figure 1 (also see Multimedia Appendix 2). Their key characteristics and findings are summarized in Table 1 [15-34]. In terms of breast cancer screening modalities, 19 articles focused on breast screening in general or mammograms in breast screening, while 1 article addressed ultrasound, which is relevant in the context of supplemental imaging for women with dense breasts. The majority of studies were from the United States (n=5), the United Kingdom (n=4), Australia (n=2), Saudi Arabia (n=1), Pakistan (n=1), France (n=1), and Korea (n=1); 4 studies were global studies. The year of publication of articles ranged from 2019 to 2023. All 20 studies were rated as moderate to high quality based on the quality assessment and met our inclusion criteria; a full quality assessment is appended in Multimedia Appendix 3.

**Table 1:** Summary of all included studies with the identification of challenges and solutions.

Study	Country	Sample population	Methods	Identification of issues and solutions across the framework							
				Reproducibility	Evidentiary standards	Technology concerns	Trust issues	Ethical concerns	Legal concerns	Societal concerns	Postadoption uncertainty
Logan et al [15]	Australia	N/A^a^	Review	Issues and solution identified	Issues and solution identified	Issues and solution identified	Issues and solution identified	Issues and solution identified	N/A	N/A	Issues and solution identified
Sufyan et al [29]	Pakistan	N/A	Review	Issues and solution identified	Issues and solution identified	N/A	Issues and solution identified	Issues and solution identified	Issues and solution identified	N/A	N/A
Al Kuwaiti et al [16]	Saudi Arabia	N/A	Review	Issues and solution identified	Issues and solution identified	Issues and solution identified	Issues and solution identified	Issues and solution identified	Issues and solution identified	Issues and solution identified	N/A
Badal et al [28]	United Kingdom	N/A	Perspective paper	Issues and solution identified	Issues and solution identified	N/A	Issues and solution identified	Issues and solution identified	N/A	Issues and solution identified	Issues and solution identified
Cushnan et al [18]	United Kingdom	N/A	Review	Issues and solution identified	Issues and solution identified	N/A	N/A	N/A	N/A	N/A	N/A
Van Nijnatten et al [24]	United Kingdom	N/A	Review	Issues and solution identified	Issues and solution identified	Issues and solution identified	N/A	N/A	N/A	N/A	N/A
Singh et al [25]	Global	N/A	Review	N/A	Issues and solution identified	Issues identified	Issues identified	Issues identified	Issues identified	Issues and solution identified	N/A
Taylor-Phillips et al [26]	United Kingdom	N/A	Review	Issues and solution identified	Issues and solution identified	Issues and solution identified	N/A	N/A	N/A	N/A	N/A
Gastounioti et al [19]	Global	N/A	Review	Issues and solution identified	Issues and solution identified	N/A	Issues and solution identified	Issues and solution identified	Issues identified	Issues and solution identified	Issues identified
Potnis et al [30]	United States	N/A	Review	Issues and solution identified	Issues and solution identified	N/A	Issues and solution identified	Issues and solution identified	Issues and solution identified	Issues and solution identified	Issues and solution identified
Hendrix et al [22]	United States	Respondents (n=66; 44% response rate) were from 6 diverse practice settings across 8 states.	Qualitative interviews	Issues identified	Issues identified	Issues identified	Issues identified	N/A	N/A	N/A	N/A
Hickman et al [23]	Global	N/A	Review	Issues identified	Issues and solution identified	Issues and solution identified	Issues and solution identified	Issues and solution identified	Issues and solution identified	N/A	N/A
Hendrix et al [21]	United States	N/A	Review	Issues identified	Issues and solution identified	Issues and solution identified	Issues identified	Issues identified	Issues identified	Issues identified	N/A
Tran et al [27]	Global	N/A	Perspective paper	Issues and solution identified	Issues and solution identified	N/A	N/A	N/A	N/A	N/A	N/A
Kim et al [31]	South Korea	N/A	Review	Issues and solution identified	Issues and solution identified	Issues and solution identified	Issues and solution identified	Issues and solution identified	N/A	N/A	N/A
Lamb et al [20]	United States	N/A	Review	Issues and solution identified	Issues and solution identified	Issues and solution identified	Issues and solution identified	Issues and solution identified	Issues and solution identified	N/A	Issues and solution identified
Bahl et al [34]	United States	N/A	Review	Issues and solution identified	Issues and solution identified	Issues and solution identified	Issues and solution identified	N/A	N/A	Issues and solution identified	N/A
Le et al [17]	United Kingdom	N/A	Review	Issues and solution identified	Issues and solution identified	Issues and solution identified	N/A	N/A	N/A	N/A	N/A
Carter et al [32]	Australia	N/A	Review	Issues and solution identified	Issues and solution identified	Issues and solution identified	Issues and solution identified	Issues and solution identified	Issues and solution identified	Issues and solution identified	N/A
Thomassin-Naggara et al [33]	France	9 experts in breast disease management convened for this consensus	Consensus paper	Issues and solution identified	Issues and solution identified	Issues and solution identified	N/A	Issues and solution identified	Issues and solution identified	N/A	N/A

^a^N/A: not applicable.

### Data Set Limitations and Bias in AI Models

Through iterative discussions, 8 themes were identified based on the reading of the studies. These encompass the challenges in the adoption of AI in breast screening: reproducibility, evidentiary standards, technology concerns, trust issues, ethical concerns, legal concerns, societal concerns, and uncertainty after the adoption of AI in breast cancer screening. Data set and validation limitations have been described by most articles [17,23,27]. Common concerns include the quality of the data set and the lack of transparency regarding the data being used for AI development [12]. Ascertainment bias may arise when cancer detection is based on decisions made by human readers instead of alternatives, such as histopathological diagnosis, impacting the reliability of ground truths used for training or evaluation of cancer detection algorithms. Sufyan et al [29] described the lack of and inconsistency in the annotation of data used to train an AI model, which can result in unreliable predictions. When AI software is trained on biased data sets, such as those that predominantly represent specific races or age groups, or exhibits inherent algorithm bias, it can lead to discriminatory results. For instance, higher breast density is common among younger and Asian women, and this increased density is linked to potential inaccuracies in AI performance during breast cancer screenings. AI systems built on mammograms that have undergone resizing, augmentation, or specific segmentation processes may struggle with accurate lesion classification due to the potential loss or distortion of original pixel information, affecting the appearance of lesions. This limitation may hinder the AI’s ability to generalize its learning to new, unseen data, resulting in poor classification performance. With regard to AI in ultrasound screening, Kim et al [31] reported concerns that high interobserver variability during the acquisition and interpretation of images may result in diagnostic inaccuracies and, thus, management discrepancies. Additionally, Gastounioti et al [19] suggested that the large variability could affect the perceived clinical applicability of AI-generated risk assessments based on mammographic evaluations, emphasizing that reproducibility, generalizability, and interpretability are fundamental principles to encourage the translation of AI into clinical practice.

### Concerns on Evidentiary Standards in AI for Breast Screening

In terms of evidentiary standards, several studies reported the quality of supporting evidence as a primary concern in determining the utility of AI in the clinical setting [21,25]. Evidentiary standards have been largely limited by the use of data-enriched data sets and small sample sizes [29,30]. Cancer-enriched data sets contain more true-positive cases than in a conventional screening setting. The risk of false positives is likely to increase in such data sets, leading to unnecessary investigations, biopsies, and anxiety. Limited, single-center studies or studies that lack clinical diversity in terms of patients of various ages, breast density, and breast cancer risks may result in poor generalizability [29,30]. The paucity of clinical validation and patient-centric outcomes was also highlighted [16,33]. Evaluation processes have primarily focused on AI’s performance rather than actual clinical outcomes for patients and health systems. Moreover, cancer detection by AI has not been shown to translate into improved health outcomes. Taylor-Phillips et al [26] suggested the use of clinically significant and relevant outcomes (eg, interval breast cancers) to evaluate the overall effect of AI, given the potential downstream effects that false positives in screening may have on the allocation of health care resources. The absence of standardized performance metrics across studies makes it challenging to compare the effectiveness of different AI models [31].

### Technological Concerns in AI Implementation

With regard to technological concerns and requirements, Thomassin-Naggara et al [33] highlighted the need for large storage capacity for massive data, while Lamb et al [20] emphasized the need to ensure compatibility of the AI system with local practice techniques and equipment. Seamless integration of AI systems with existing health care information systems, such as picture archiving and communication systems [43] and electronic health records, is critical for effective collaboration and data sharing. AI algorithms, especially deep learning models, often require significant computational power. The technical expertise to develop and maintain information technology infrastructure [21] is also necessary to support AI systems and ensure scalability in breast cancer screening programs. Additionally, radiologists will require training to understand the appropriate use of various tools and their limitations [21,23,34]. In terms of the AI user interface, through qualitative interviews, Hendrix et al [22] found that 26%-33% of radiologists were deterred if the AI features did not align with their preferences, highlighting the need for increased collaboration between radiologists and technical professionals to bridge this gap.

### Trust Issues Among Radiologists and Clinicians

Pertaining to trust issues, Lamb et al [20] described the variability in the level of trust in AI among radiologists, nonradiologist clinicians, and patients. Similarly, Hendrix et al [22] reported that physicians remain wary of the use of AI for unsupervised or partially supervised image interpretation. Carter et al [32] and Bahl [34] postulated that this is related to the tendency of AI to recommend individualized decisions that are not explainable, rather than providing general recommendations at a population level, as in a conventional screening setting. The term “black box” refers to an AI system whose internal workings are not transparent or easily understandable [30]. Intellectual property clauses protecting proprietary sources fuel the lack of transparency by limiting the distribution and sharing of the AI source code and architecture [35]. With limited human involvement in the decision-making process and a lack of clear explanation for how AI systems arrive at their recommendations, there is significant skepticism and reduced trust in the decisions made with the help of AI [25,32].

### Ethical Concerns in AI for Breast Cancer Screening

Ethical concerns are also significant in the context of AI in health care. Biases embedded in training data can be perpetuated by AI algorithms, leading to discrepancies in breast cancer screening accuracy among various demographic groups [12,15,30]. Existing inequalities may be exacerbated by biased data input into the algorithm, creating a negative feedback loop that further intensifies these disparities [23]. Badal et al [28] highlighted that adopting AI technologies in health care necessitates specialized skills, knowledge, and resources, including trained personnel who can develop, operate, and maintain these systems. For disadvantaged populations, who often face obstacles such as limited health care resources and a shortage of trained professionals, these requirements can present considerable challenges. Consequently, the very systems intended to enhance health care outcomes may not reach or be effectively utilized by those who need them most, thereby perpetuating existing inequities in health care access and quality. Furthermore, Al Kuwaiti et al [16] discussed the lack of transparency and explainability of AI, which raises ethical questions regarding accountability. Lamb et al [20] and Carter et al [32] pointed out the potential for conflicts of interest, particularly between clinicians and developers, which could influence the implementation of AI systems. Clinicians may become “liability sinks,” where they bear the legal consequences of AI-related errors without having full control or understanding of the AI’s decision-making processes [36]. Both Badal et al [28] and Carter et al [32] stress the importance of shared decision-making, asserting that women undergoing screening should have the autonomy to decide whether to incorporate AI-enabled screening into their health care.

### Legal Challenges in AI for Breast Screening

With regard to legal challenges, data breaches in the context of AI are particularly concerning, as they refer to incidents where unauthorized individuals or entities gain access to sensitive and confidential information processed or stored by AI systems [37]. These breaches can have significant consequences, ranging from privacy violations to identity theft and other forms of cybercrime. Another legal challenge is the determination of legal liability if AI systems in breast screening make errors leading to misdiagnosis or patient harm [23]. Determining responsibility for algorithmic errors—whether it lies with the developers, health care providers, or both—raises complex legal questions. Potnis et al [30] stated that the Food and Drug Administration’s role is to maintain a minimum threshold for AI product approval and suggested that interested parties have responsibilities regarding how AI is being adopted. Carter et al [32] described a regulatory vacuum for AI-related technologies. The absence of clear regulations can create uncertainty for stakeholders about the legal boundaries associated with AI adoption.

### Impact of AI on Professional Development and Society

Concerns were also raised regarding the broader impact of AI on professional development and society. There are fears that AI may lead to job displacement or increased dependency among radiologists, particularly if it assumes responsibility for interpreting normal mammograms [25]. Similarly, Carter et al [32] and Hickman et al [23] warned that overreliance on AI in such tasks could diminish radiologists’ diagnostic skills or reduce their familiarity with normal imaging, potentially resulting in the oversight of important clinical nuances. Other potential biases include automation bias, where readers may be overly influenced by AI-generated decisions, leading to overcommitment to false positives or omission of other abnormalities. Additionally, the “anchoring effect” may occur—once markers indicating potential malignancy are placed on an image, they can unduly influence the reader’s judgment and subsequent decision-making [17]. Concerns have been raised that the increasing reliance on AI may undermine essential human elements in health care, such as nuanced clinical judgment and collaborative care, potentially affecting the quality of patient experience and outcomes [16]. Radiologists may also resist the adoption of AI in breast cancer screening due to doubts about the reliability of the technology, fears of job displacement, and skepticism about whether AI can truly surpass human expertise [20].

### Postadoption Uncertainty and Stakeholder Impact

There is also a prevailing sense of uncertainty following AI adoption, as models may be exposed to diverse and evolving clinical conditions that were not adequately represented in their training data. Logan et al [15] highlighted the potential for unintended consequences of AI implementation, which could impact various stakeholders, including patients, radiologists, and health care institutions. These adverse events may include misdiagnoses, false positives, or false negatives, each of which can carry significant clinical and emotional consequences for patients.

### Proposed Recommendations

**Figure 2:**
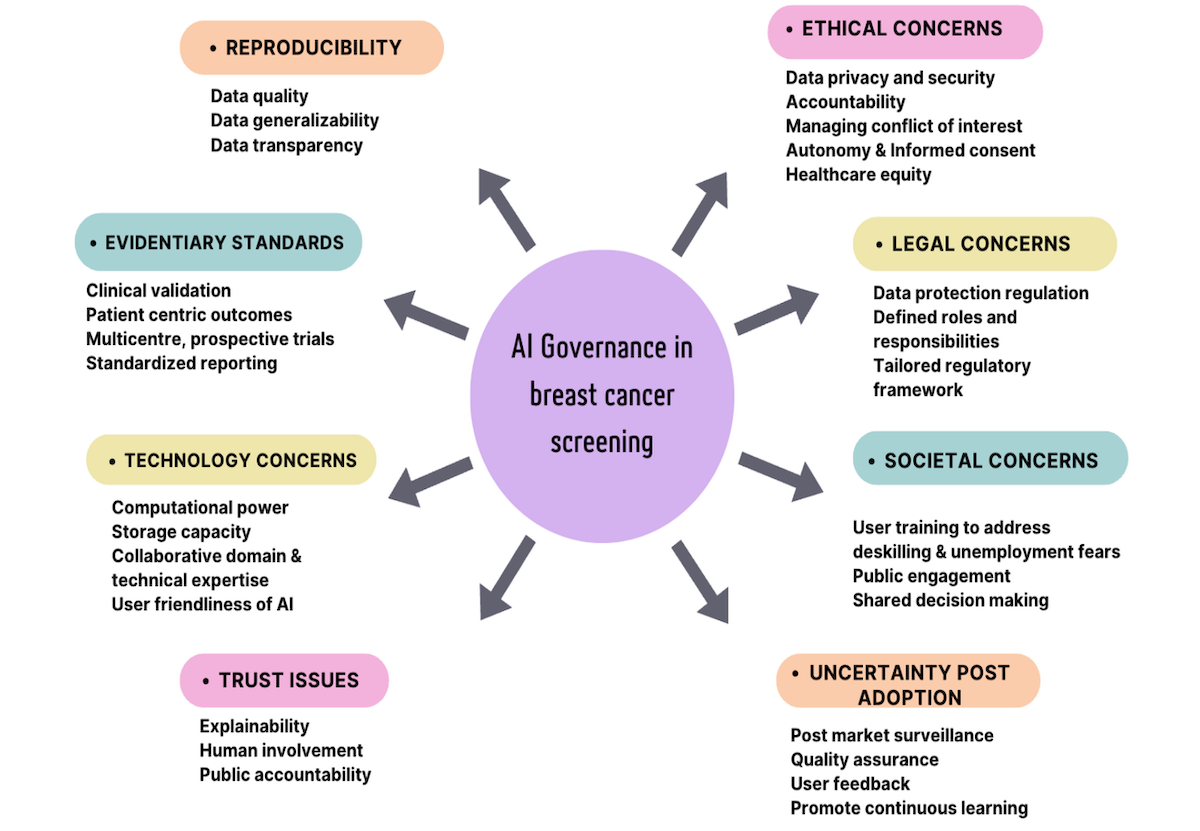
Proposed governance framework for the safe and effective integration of AI in breast cancer screening programs. AI: artificial intelligence.

Alongside the identified themes, we propose practical and innovative solutions guided by the CFIR, as illustrated in Figure 2.

**Figure 3:**
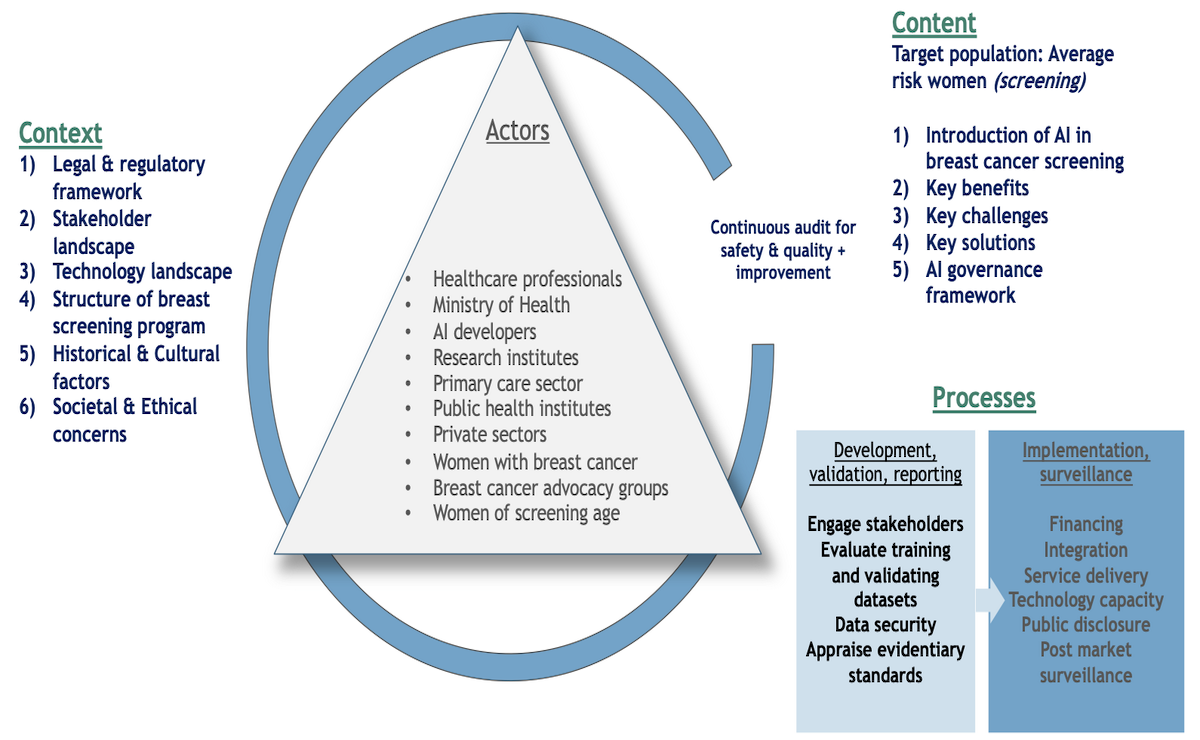
Policy triangle illustrating the critical actors, contextual factors, and implementation processes necessary for developing and executing effective AI governance frameworks in breast cancer screening. AI: artificial intelligence.

Using Singapore and our institutional experience as a case study, we outline 3 key components (actors, context, and processes) that are critical for the development and implementation of policies related to AI in breast cancer screening. These components are presented sequentially in Figure 3. The establishment of a tailored AI governance framework, specifically for mammography, is essential to ensure the safe, accurate, and ethical integration of AI technologies into clinical workflows.

## Discussion

### Principal Findings

Based on a review of 20 articles, this study identifies the multifaceted challenges associated with the implementation of AI in breast cancer screening and proposes a governance framework guided by the CFIR. The findings align with the study’s objective to explore the barriers and facilitators to AI adoption in breast screening programs. Key themes that emerged are issues of reproducibility, evidentiary standards, technological requirements, trust and transparency, ethical and legal considerations, societal impacts, and postadoption uncertainty. These insights offer practical guidance for optimizing AI integration in complex, real-world health care environments.

While AI has the potential to enhance mammogram interpretation and improve early breast cancer detection [38], the main findings of this study underscore the complex challenges associated with its adoption. Key themes identified are issues related to reproducibility, evidentiary standards, technological concerns, trust and transparency, ethical considerations, legal challenges, societal impacts, and uncertainty following AI implementation.

The innovation characteristics domain of the CFIR underscores the potential advantages of AI, particularly in enhancing the accuracy and efficiency of mammogram interpretation. When mapped to CFIR constructs, adaptability and complexity emerge as critical factors influencing the successful integration of AI into breast cancer screening. The perceived complexity of AI, especially regarding how seamlessly it fits into existing clinical workflows, significantly affects its adoption and implementation. AI systems depend on complex algorithms, large data sets, and real-time data processing, all of which contribute to their perceived complexity, particularly when radiologists must balance trust in AI outputs with their own clinical judgment [39,40]. This complexity is further amplified by the need for seamless integration with hospital information technology infrastructure, including electronic health records and imaging systems. If AI tools lack adaptability across diverse health care environments or demand substantial technical support, integrating them into existing workflows becomes significantly more challenging.

The strength and quality of evidence are critical to the successful adoption and integration of AI in breast cancer screening. This encompasses the perceived credibility, reliability, and validity of the evidence supporting AI’s use. By incorporating gold-standard references, such as histopathology results, ascertainment bias can be minimized, thereby enhancing the reliability of AI algorithms in detecting breast cancer [41]. As Thomassin-Naggara et al [33] noted, the integration of such standards strengthens training data and provides a solid foundation for the broader application of AI in clinical practice. Standardizing the AI training and annotation process is essential for the accurate identification and classification of mammographic features, which are critical for early cancer detection. Without standardization, inconsistent labeling can introduce biases that undermine the performance of the algorithm. Regular data quality control measures are necessary to address discrepancies, while continuous monitoring and feedback loops are vital to correct data errors and ensure the long-term reliability of AI systems.

The lack of clear and consistent regulations for AI in health care, particularly in mammography, poses a significant barrier to its adoption. Many health care institutions are reluctant to integrate AI tools without a well-defined framework that outlines legal responsibilities, risk management protocols, and data handling standards essential for the safe deployment of these technologies. Regulatory uncertainty can cause delays in the adoption of AI technologies in breast cancer screening. While not explicitly addressed in the studies reviewed, it is likely that financial incentives, such as reimbursement policies for AI-assisted imaging, could facilitate AI integration into clinical practice. In health care systems that use outcome-based payment models, the added value of AI in enhancing diagnostic accuracy may help justify the initial investment in AI technologies. Conversely, in systems lacking clear reimbursement pathways for AI tools, health care institutions may be reluctant to invest in the upfront costs of these technologies [37]. The outer setting also plays a crucial role in understanding patients’ values and beliefs. Engaging women in discussions about the role of AI in breast cancer screening can foster greater acceptance and ensure that these innovations meet their needs effectively [30]. A study involving 800 women undergoing breast cancer screening revealed that 88% of participants had a positive view of AI in medicine, with 51% reporting some knowledge of AI [42]. Notably, the majority of women preferred AI to serve as a second reader, emphasizing the importance of human oversight in the diagnostic process [42].

In the inner setting, robust digital infrastructure and specialized technical expertise are essential resources for the successful integration of AI. Equally important is fostering communication and collaboration between breast radiologists, data scientists, and engineers. Aligning the missions and goals of these groups ensures that AI algorithms remain clinically relevant, user-friendly, and adhere to the ethical standards of health care. A shared dialogue fosters a deeper understanding of radiologists’ needs and allows technical experts to tailor AI functionalities to better enhance clinical workflows. Research by Hendrix et al [22] highlighted that radiologists’ acceptance of AI technologies is strongly influenced by how well these systems meet their functional requirements and performance expectations. Transparency is a key element in fostering this relational connection. For example, providing radiologists with visual feedback, such as superimposed heat maps highlighting suspicious areas on mammograms, can improve their understanding of AI-driven predictions and enhance diagnostic accuracy. Additionally, cultural factors within the hospital setting, such as radiologists’ openness to adopting AI tools, are crucial in determining the success of AI implementation. In institutions where traditional diagnostic practices dominate, cultural resistance to AI adoption may arise, particularly among senior radiologists who perceive it as a threat to job security or professional expertise [43]. Furthermore, the urgency for change, driven by rising workloads and the need to reduce screening errors, can either accelerate or delay the adoption of AI technologies.

Comprehensive training programs are essential in building confidence among radiologists when using AI tools. Such initiatives can help alleviate concerns about job displacement and deskilling, allowing radiologists to view AI as a valuable resource rather than a threat. When radiologists trust the algorithms and the quality of data behind AI, they are more likely to integrate this technology into breast screening practices. To foster trust, organizations should emphasize transparency by providing insights into how AI systems function, including details about the data sets used and the validation processes involved. A clear understanding of AI tool development and data set transparency can help radiologists view these systems as reliable assistants in their diagnostic workflow. Furthermore, validating AI algorithms on diverse and representative data sets, particularly local ones, enhances the generalizability and effectiveness of cancer detection algorithms in real-world applications. As noted by Hickman et al [23], a trustworthy algorithm that delivers clear, consistent, and reproducible results with minimal ambiguity in decision-making is crucial for building confidence in AI systems. Additionally, opinion leaders, such as respected clinicians or thought leaders in the field of breast cancer, can significantly shape the attitudes and behaviors of their colleagues. Their endorsement of AI technologies can help reduce resistance and foster a positive perception among radiologists and other health care professionals.

A systematic approach to implementation involves several key phases. Initially, a retrospective evaluation of AI software using a large, representative data set should be conducted for benchmarking purposes. This should be followed by prospective assessments in clinical settings, as advocated by researchers such as Le et al [17] and Taylor-Phillips et al [26]. This stepwise approach is crucial, especially considering the resource-intensive nature of prospective studies, which can hinder the broader adoption of AI in mammography practices. Moreover, alternative methodologies, such as virtual clinical trials, present a practical solution to the resource limitations associated with traditional randomized controlled trials [19]. These trials leverage simulated environments to assess the performance of AI algorithms without the logistical and ethical complexities associated with live patients. Such virtual assessments can effectively evaluate how well AI identifies interval cancers that may be missed by human readers, offering a scalable and efficient way to benchmark AI algorithms. In addition to developing diverse data sets, simulation models can project AI’s long-term effects on breast screening outcomes, including morbidity and mortality rates [30,44]. These models assist stakeholders in understanding the potential benefits and risks associated with AI integration in clinical practice, thus guiding future implementation efforts.

After implementation, ensuring consistent evaluation and benchmarking is crucial for integration. Establishing clear metrics and adhering to preclinical reporting guidelines such as TRIPOD-AI (Transparent Reporting of a Multivariable Prediction Model for Individual Prognosis or Diagnosis—Artificial Intelligence), as well as clinical reporting guidelines such as STARD-AI (Standards for Reporting of Diagnostic Accuracy Studies—Artificial Intelligence) [45], DECIDE-AI (Development and Evaluation of Complex Interventions for Decision-Making in AI) [46], CONSORT-AI (Consolidated Standards of Reporting Trials—Artificial Intelligence) [47], and SPIRIT-AI (Standard Protocol Items: Recommendations for Interventional Trials—Artificial Intelligence) [48], is essential for conducting postimplementation studies rigorously and transparently. The commitment to quality enhances the credibility of AI applications in breast cancer detection and supports the seamless integration of AI into existing health care workflows [49]. Postmarket surveillance emerges as a vital component for maintaining the safety, efficacy, and ongoing relevance of AI technologies in breast screening [30]. Logan et al [15] advocated for quality assurance checks to ensure AI systems meet safety and performance standards. Regular performance reviews focused on key cancer-related outcomes, such as sensitivity, specificity, rate of early cancer detection, and interval breast cancer rates, will be required for refining AI systems and ensuring their alignment with clinical goals. This monitoring process allows for the early detection and rectification of any issues that could compromise patient outcomes. Badal et al [28] emphasized that AI algorithms should be designed for ongoing evaluation, learning, and iterative updates to address evolving clinical needs and integrate new scientific knowledge. By continuously adapting based on real-world feedback, postmarket surveillance helps to mitigate unforeseen errors, ensuring AI technologies remain both effective and safe in breast cancer screening [49].

The creation of a tailored AI governance framework for breast cancer screening, particularly in mammography, is crucial to ensure the technology’s safe, accurate, and ethical integration into clinical workflows. Ultimately, this governance framework can guide the responsible implementation of AI in breast mammography, enhancing early detection, reducing missed cancers, and improving patient outcomes, all while maintaining a human-centered approach to breast cancer care. Reflecting and evaluating are essential to continuous improvement, and further refinement will require a collaborative and iterative process. Policy makers must tailor their strategies to ensure that the AI adoption framework is responsive to the unique challenges and opportunities in each country [50].

This systematic review lays the groundwork for developing a comprehensive AI governance framework for breast cancer screening. Given the burgeoning interest in this field, our methodology was both timely and appropriate for gathering the necessary information. The identified themes are interdependent, highlighting the real-world complexities of AI implementation. For example, issues with reproducibility directly impact evidentiary standards, while ethical concerns about data breaches can lead to significant legal ramifications. The rapidly evolving nature of AI necessitates regular reviews of new publications to remain current with the latest developments. Future efforts should incorporate additional methodologies such as focused group discussions with stakeholders, real-world case study analyses, and international collaborations [50]. These approaches can provide deeper insights and more robust contributions to the development of an effective AI governance framework.

Nonetheless, this study is not without limitations. First, the review focused only on English-language articles, potentially omitting relevant findings in other languages. Second, the heterogeneity of included studies and the lack of standardized reporting in AI research may have influenced the synthesis and applicability of findings. Third, while the CFIR provides a comprehensive lens, it does not prioritize specific domains, which may limit its practical implementation without further contextual tailoring. Last but not least, this review also uncovered significant imbalances in the available literature across different domains. While there is ample discussion on reproducibility, evidentiary standards, and technological concerns, there is a relative paucity of studies addressing legal issues and postadoption uncertainty. This gap underscores the need for future research to delve more deeply into the legal and ethical dimensions of AI use in breast cancer screening.

In summary, this study highlights the critical need for robust governance frameworks to address the complexities of integrating AI into breast cancer screening. While AI has the potential to improve diagnostic accuracy and efficiency, its broader implications include promoting equitable health care delivery, strengthening patient trust, and supporting the ethical development of AI technologies. Policy makers, clinicians, and AI developers must work collaboratively to establish adaptable and transparent systems that prioritize patient safety and societal benefits. Future research should focus on real-world case studies, longitudinal assessments, and cross-disciplinary collaborations to effectively refine and implement these governance strategies.

### Conclusions

This systematic review identified key challenges in the implementation of AI for breast cancer screening, including the need for consistent application, robust evidence, and technological advancements. Using the CFIR, the review offered a structured approach to address these barriers, promoting trust, ethical governance, and equitable access. These findings provide a comprehensive framework for integrating AI into patient-centered breast cancer screening programs in a safe and effective manner.

## Appendix

Multimedia Appendix 1 Search strategy.

Multimedia Appendix 2 PRISMA checklist.

Multimedia Appendix 3 Quality assessment of included studies evaluating AI in breast cancer screening. AI: artificial intelligence.

## Abbreviations

AI: artificial intelligence
CFIR: Consolidated Framework for Implementation Research
CONSORT-AI: Consolidated Standards of Reporting Trials—Artificial Intelligence
DECIDE-AI: Development and Evaluation of Complex Interventions for Decision-Making in AI
PRISMA: Preferred Reporting Items for Systematic Reviews and Meta-Analyses
PROSPERO: International Prospective Register of Systematic Reviews
SPIRIT-AI: Standard Protocol Items: Recommendations for Interventional Trials—Artificial Intelligence
STARD-AI: Standards for Reporting of Diagnostic Accuracy Studies—Artificial Intelligence
TRIPOD-AI: Transparent Reporting of a Multivariable Prediction Model for Individual Prognosis or Diagnosis—Artificial Intelligence

## Data Availability

The data analyzed in this study are available from the corresponding author on reasonable request. Additionally, all included studies were sourced from publicly available databases such as PubMed, Embase, and Web of Science. Detailed search strategies and inclusion criteria are provided in Multimedia Appendices 1-3. The corresponding author had full access to all data used in the study and takes the final responsibility for the decision to submit this article for publication.
