# Supplementary figures and images for: Challenges in Implementing Artificial Intelligence in Breast Cancer Screening Programs: Systematic Review and Framework for Safe Adoption
Source: J Med Internet Res. 2025 May 15;27:e62941. doi: 10.2196/62941 (PMC12123233; doi:10.2196/62941)

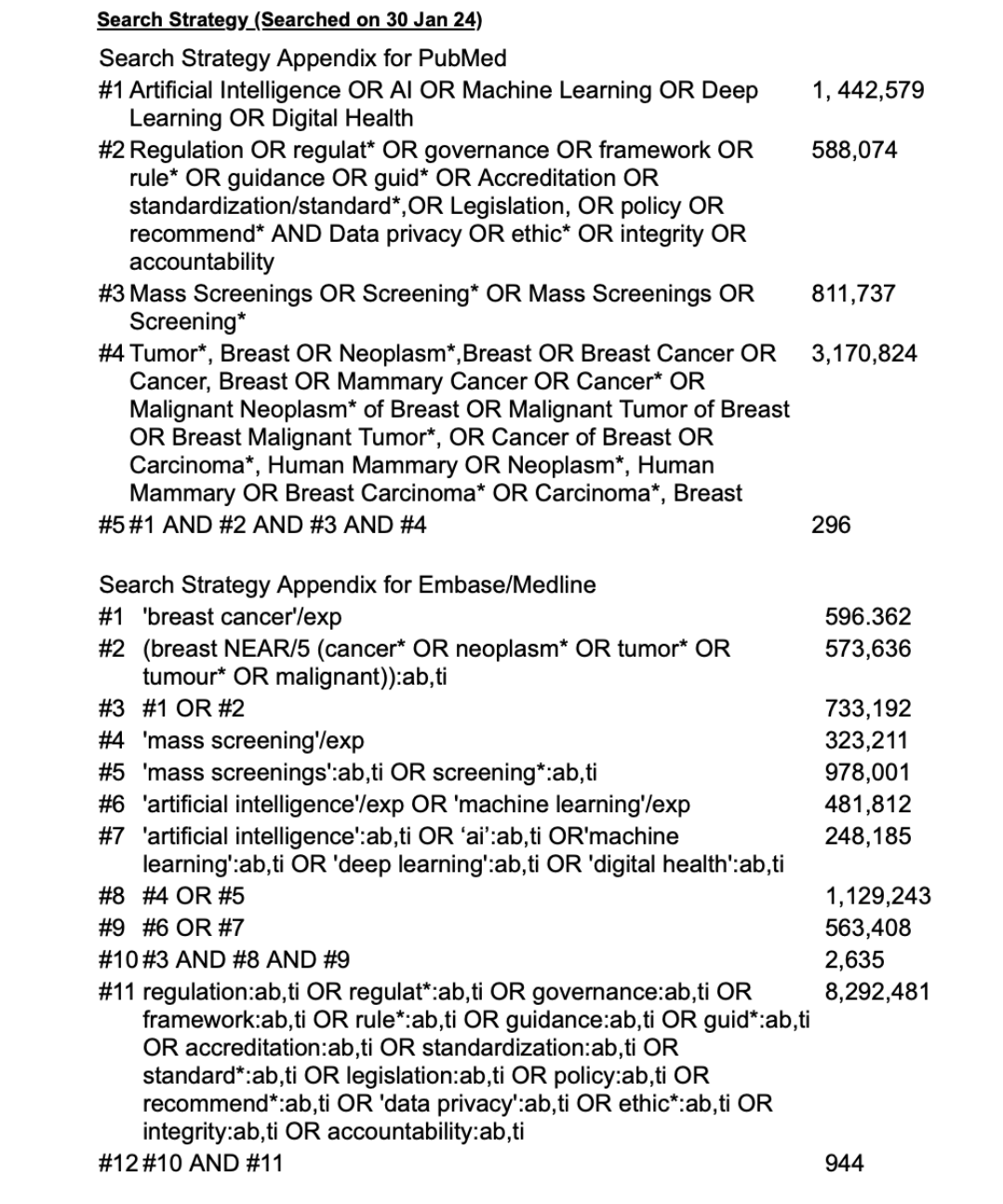

Supplement: Multimedia Appendix 1 [file jmir_v27i1e62941_app1.png]

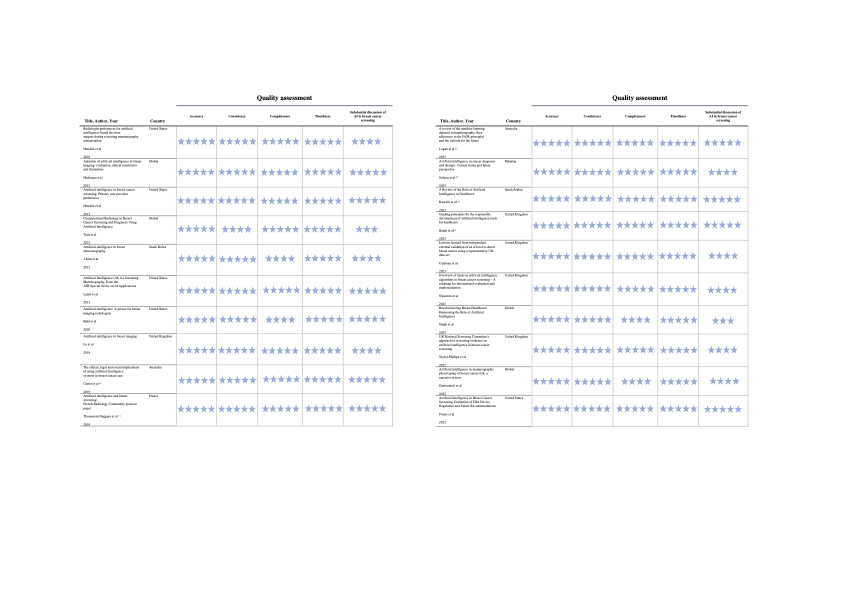

Supplement: Multimedia Appendix 3 [file jmir_v27i1e62941_app3.png]
